# Role of the IFN I system against the VHSV infection in juvenile Senegalese sole (*Solea senegalensis*)

**DOI:** 10.1186/s13567-015-0299-4

**Published:** 2016-01-08

**Authors:** Daniel Alvarez-Torres, Ana M. Podadera, Julia Bejar, Isabel Bandin, M. Carmen Alonso, Esther Garcia-Rosado

**Affiliations:** Departamento de Microbiología, Facultad de Ciencias, Universidad de Málaga, 29071 Málaga, Spain; Departamento de Genética, Facultad de Ciencias, Universidad de Málaga, 29071 Málaga, Spain; Departamento de Microbiología, Instituto de Acuicultura, Universidad de Santiago de Compostela, 15782 Santiago de Compostela, Spain; Departamento de Bioquímica y Biología Molecular, Universidad de Oviedo, Oviedo, Spain

## Abstract

Senegalese sole is susceptible to marine VHSV isolates but is not affected by freshwater isolates, which may indicate differences regarding virus-host immune system interaction. IFN I induces an antiviral state in fish, stimulating the expression of genes encoding antiviral proteins (ISG). In this study, the stimulation of the Senegalese sole IFN I by VHSV infections has been evaluated by the relative quantification of the transcription of several ISG (*Mx, Isg15* and *Pkr*) after inoculation with marine (pathogenic) and freshwater (non-pathogenic) VHSV isolates. Compared to marine VHSV, lower levels of RNA of the freshwater VHSV induced transcription of ISG to similar levels, with the *Isg15* showing the highest fold induction. The protective role of the IFN I system was evaluated in poly I:C-inoculated animals subsequently challenged with VHSV isolates. The cumulative mortality caused by the marine isolate in the control group was 68%, whereas in the poly I:C-stimulated group was 5%. The freshwater VHSV isolate did not cause any mortality. Furthermore, viral RNA fold change and viral titers were lower in animals from the poly I:C + VHSV groups than in the controls. The implication of the IFN I system in the protection observed was confirmed by the transcription of the ISG in animals from the poly I:C + VHSV groups. However, the marine VHSV isolate exerts a negative effect on the ISG transcription at 3 and 6 h post-inoculation (hpi), which is not observed for the freshwater isolate. This difference might be partly responsible for the virulence shown by the marine isolate.

## Introduction

The type I interferon (IFN I) promotes an antiviral state by inducing the transcription of numerous interferon stimulated genes (ISG), such as *Mx*, interferon-stimulated gene 15 (*Isg15*), and the protein kinase R (*Pkr*) genes [1], which will be considered as markers of the Senegalese sole (*Solea senegalensis*) IFN I activity in this study.

Mx proteins are large GTPases involved in intracellular membrane remodelling and intracellular trafficking [2]. In teleosts, the expression of these proteins in response to polyinosinic-polycytidylic acid (poly I:C) or viral infection, as well as their virus-specific antiviral activity, have been widely demonstrated [1, 3, 4]. The antiviral activity of these proteins depends on the interaction between the Mx protein and specific viral target proteins, inhibiting the viral genome synthesis or the viral particle assembly [3, 5].

ISG15 are ubiquitin-like proteins containing two tandem repeats of ubiquitin-like domains. In mammals, these proteins can conjugate to either, cellular or viral target proteins, via the C-terminal LRLRGG sequence (ISGylation), which is controlled by a series of IFN-inducible enzymes [6, 7]. However, unlike ubiquitin, ISGylated proteins are not degraded in the proteasome [8]. In addition to their antiviral activity, ISG15 proteins seem to be involved in regulating the IFN I signalling [9, 10]. In teleosts, *Isg15* gene transcription has been studied in different species [11–15], determining that fish ISG15 proteins contain the critical C-terminal glycine residues, which suggests that they could conjugate to target proteins and have antiviral activity similar to their mammalian counterparts. In fact, the ISG15 antiviral activity has been demonstrated in several fish species [12, 14].

PKR proteins are involved in many cellular processes, including cell proliferation and cell growth, apoptosis, and tumor suppression. In mammals, PKR is activated by phosphorylation triggered by double-stranded RNA (dsRNA). Once activated, PKR phosphorylates the eukaryotic initiation factor 2 (elF-2α), causing the inhibition of protein synthesis [16]. *Pkr* genes have been studied in diverse fish species to date [17–22], and their transcription after poly I:C inoculation has been reported in rock bream (*Oplegnatus fasciatus*) spleen and fugu (*Takifugu rubripes*) leucocytes [20, 21]. However, the antiviral mechanism of fish PKR has only been described in Japanese flounder (*Paralichthys olivaceus*) embryonic cells, in which the over-expression of *Pkr* increases elF-2α phosphorylation [19].

Viral Haemorrhagic Septicaemia Virus (VHSV) is the causal agent of the viral haemorrhagic septicaemia (VHS), an important disease affecting farmed salmonid species. However, the occurrence of VHSV in wild marine fish has led to the conclusion that the virus is enzootic in the marine environment and endemic in northern European waters [23]. The existence of genetic links among VHSV isolates recovered from wild fish and isolates responsible for epizootics in farmed turbot has been demonstrated [24]. Furthermore, VHSV has been recently detected in wild fish caught in southern European coastal waters [25]. Therefore, the existence of this marine VHSV reservoir may represent a potential risk for farmed Senegalese sole, which has been demonstrated to be susceptible to VHSV by experimental infection [26].

In Senegalese sole, the IFN I system has only been studied after poly I:C treatment or the inoculation with the Infectious Pancreatic Necrosis Virus (IPNV), showing antiviral activity against this viral infection [27]. In addition, the only ISG characterized in this fish species to date is *Mx*. Thus, the Senegalese sole Mx protein (SsMx) shows in vitro antiviral activity against VHSV [28], and this virus activates the *SsMx* promoter in rainbow trout (*Oncorhynchus mykiss*) gonad (RTG-2) cells [29]; however, there is no information about the in vivo response of the Senegalese sole IFN I system to VHSV infections.

In this study, the activity of the IFN I system of juvenile Senegalese sole has been evaluated by measuring the transcription of *Mx*, *Isg15* and *Pkr,* as markers of the IFN I activation, in response to poly I:C and infections with VHSV isolates pathogenic and non-pathogenic to sole. Additionally, the protection conferred by the IFN I system against both VHSV infections has been tested by the stimulation with poly I:C prior to VHSV inoculations.

## Materials and methods

### Viruses and cell culture conditions

Two VHSV isolates were used: (1) VHSV genotype III (SpSm-IAusc2897, marine isolate obtained from turbot (*Scophthalmus maximus*), pathogenic (P) to Senegalese sole) [26, 30], and (2) VHSV genotype I (DK-F1), reference strain [31, 32] (freshwater isolate obtained from rainbow trout, non-pathogenic (NP) to Senegalese sole) kindly provided by Dr. Olesen (National Veterinary Institute, Arhus, Denmark). Viruses were propagated on the epithelioma papolosum cyprini (EPC) cell line. EPC cells were grown on 75-cm^2^ flasks (Nunc) at 25 °C in Leibovitz’s medium (L15, Lonza) supplemented with 10% foetal bovine serum (FBS, Lonza), 100 IU/mL penicillin, and 0.1 mg/mL streptomycin (Lonza) until semiconfluence prior to virus inoculation. Inoculated EPC cells were maintained in L15 medium with 2% FBS, 100 IU/mL penicillin, and 0.1 mg/mL streptomycin at 15 °C, and they were monitored until cytopathic effect (CPE) emergence. Supernatants were then collected, centrifuged at 5000×*g* for 10 min at 4 °C, and the resulting viral suspensions were titrated by the endpoint dilution method on 96-well plates. The 50% tissue culture infective dose (TCID_50_) was estimated by the method of Reed and Muench [33]. Both virus isolates were stored at −80 °C until use.

### Fish challenges

Juvenile Senegalese sole specimens (between 9.5 and 11.5 g) were acclimatized for 15 days at the facilities of the University of Santiago de Compostela (Spain). Fish used in this study were treated according to the Spanish directive (RD 53/2013, BOE no. 34). Fish were maintained in 125-L aquaria with aeration under stable temperature (16 °C ± 0.5) and salinity (36–37 g/L) conditions. Prior to challenge, 10 fish were analyzed by PCR in order to discard previous infections with IPNV, VHSV or the Nervous Necrosis Virus (NNV) according to the methodology previously described [34–37]. All tests were negative (data not shown).

**Figure 1:**
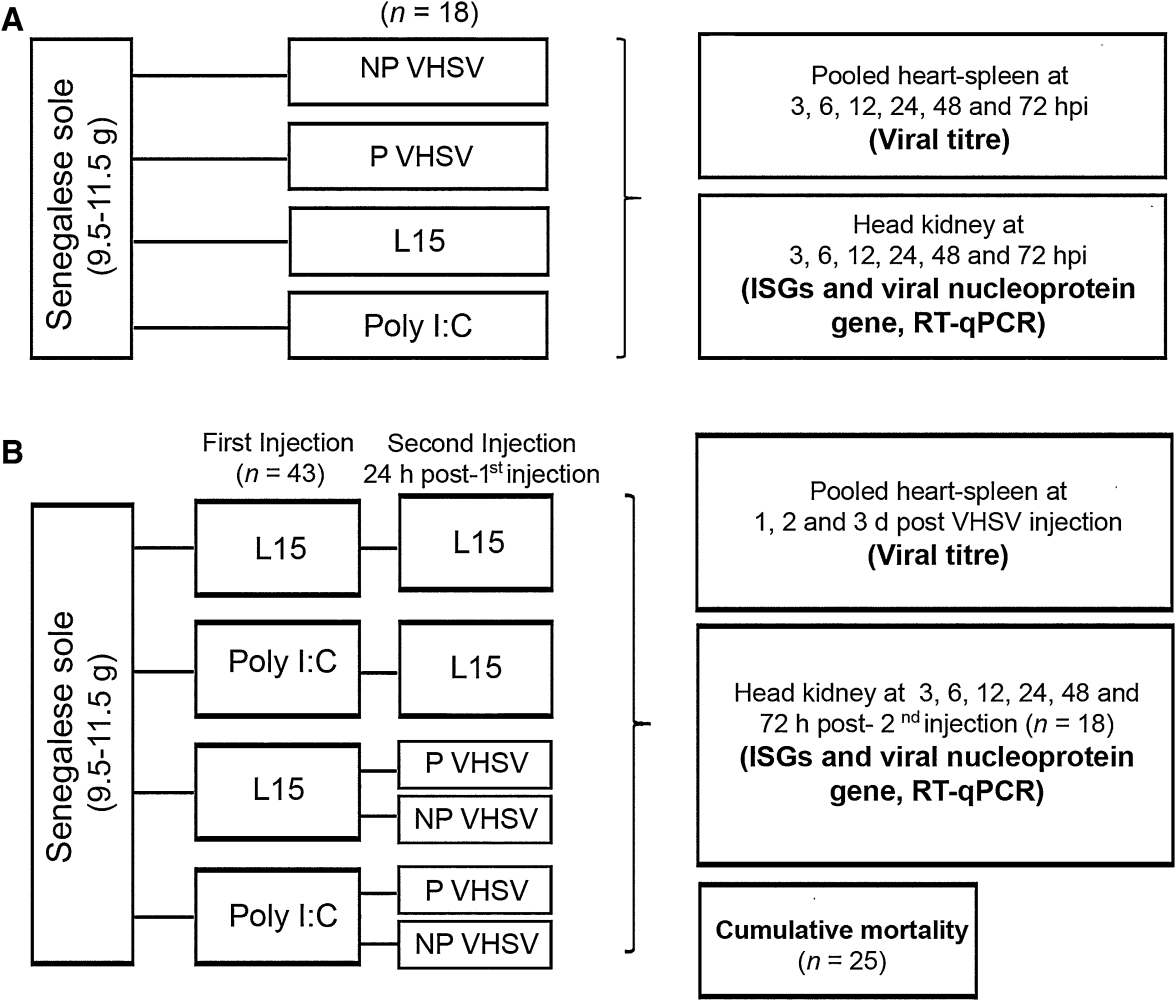
**Experimental design. A** Induction of ISG transcription by VHSV isolates pathogenic (P) or non-pathogenic (NP) to sole. L15 and poly I:C-inoculated fish were negative and positive controls, respectively. **B** Induction of an antiviral state by poly I:C. First injection was with L15 or poly I:C. Second injection (24 h after first inoculation) was with L15 (negative control) or pathogenic or non-pathogenic VHSV isolates. Cumulative mortality was determined. In both challenges, ISG transcription was quantified and viral multiplication was also evaluated by viral genome quantification and viral titration.

For the analysis of the *Mx*, *Isg15* and *Pkr* transcription in response to VHSV infections, Senegalese sole specimens were distributed in four groups (*n* = 18 per group, Figure 1A): (1–2) virus-inoculated groups (inoculated with the freshwater (NP) or marine (P) VHSV isolate, 10^4^ TCID_50_/fish), (3) negative control group (inoculated with L15), (4) positive control group (inoculated with poly I:C, 15 mg/Kg in L15). All the inoculations were performed by intraperitoneal injection (IP) in 0.1 mL (final volume).

Three animals per group were sacrificed by anaesthetic overdose (MS-222, Sigma) at 3, 6, 12, 24, 48 and 72 hpi. Head kidney, and pooled spleen and heart were aseptically collected and individually processed as indicated below. Head kidney samples were stored at −80 °C in RNA later solution (Ambion) until RNA extraction. Pooled spleen-heart samples were stored at −20 °C until virus quantification.

The putative protection conferred by the IFN I system elicited by poly I:C against the marine and freshwater VHSV isolates was also evaluated. To fulfil this objective, the following six groups (*n* = 43 per group) were considered: (1) L15 + L15 group (first and second inoculation with L15), (2) poly I:C + L15 group (first inoculation with poly I:C, second inoculation with L15), (3–4) L15 + VHSV groups (first inoculation with L15, second inoculation with the VHSV isolates), (5–6) poly I:C + VHSV groups (first inoculation with poly I:C, second inoculation with the VHSV isolates) (Figure 1B). All the inoculations were performed by IP injection (0.1 mL, final volume). Second inoculation was always 24 h after the first inoculation. Poly I:C concentration was 15 mg/Kg. The viral inoculum was 2 × 10^5^ TCID_50_/fish.

Head kidneys were collected from fish at 3, 6, 12, 24, 48 and 72 h after second injection. Pooled spleen-heart were sampled at 1, 2 and 3 days post-2nd inoculation. Three animals were sacrificed per group, and samples were individually processed. Spare fish (*n* = 25) were maintained for 30 days in order to record the cumulative mortality. Dead fish were processed to calculate the viral titer.

### Tissue processing for viral titration

Pooled spleen-heart from sampled and dead fish, were homogenized (10% w/v) in Earle’s medium (Hyclone) supplemented with 0.1 mg/mL gentamicin (Lonza), 100 IU/mL penicillin, and 0.1 mg/mL streptomycin. Homogenates were centrifuged at 600×*g* for 20 min at 4 °C, and the resulting supernatants were collected and incubated for 12 h at 4 °C. Treated supernatants were stored at −80 °C until titration on EPC cells by the TCID_50_ method. Sample viral titers were calculated in triplicate and data were log-transformed. Mean values were statistically analyzed by one-way analysis of variance (ANOVA) and Bonferroni test. Differences were considered statistically significant when *p* < 0.05.

### RNA isolation and cDNA synthesis

For RNA isolation, organs in RNA later were thawed, and the tissues were homogenized (10% w/v) in L15 supplemented with 2% FBS, 100 IU/mL penicillin and 0.1 mg/mL streptomycin, using the T10 basic Ultra-Turrax (IKA). Total RNA isolation was carried out on 250 μL of tissue homogenate using the TRI Reagent (Sigma), according to the manufacturer’s instructions. Final RNA concentration was measured at 260 nm with the nanodrop system (ND-1000), and RNA quality was checked by electrophoresis. RNA was stored at −80 °C until use.

Total RNA was treated with DNase (Roche) according to the manufacturer’s instructions. Complementary DNA (cDNA) synthesis was accomplished using 500 ng of RNA, the SuperScript II Reverse Transcriptase (Invitrogen) and random hexamer primers in a 20-μL reaction mix according to the manufacturer’s instructions. cDNA concentration was determined at 260 nm with the nanodrop system. cDNA was stored at −20 °C until use.

### Quantification of ISG transcription and viral RNA

The transcription of the *Mx*, *Isg15*, and *Pkr* genes, as well as the relative fold change values of the viral nucleoprotein (N) RNA, were quantified by SYBR Green real-time PCR protocols using specific primers (Table 1). The specificity of these primers was determined by melting curve analyses and sequencing of each amplified product (Genetic Analyzer ABI PRIMS 3130, Applied Biosystems) (data non-shown). The ubiquitin (*Ubq*) and the ribosomal protein S4 (*Rps4*) were used as housekeeping genes (Table 1).

**Table 1:** **Primers used in this study.**

Gene	Primer name	Primer sequence (5′–3′)	Amplicon size (bp)
*Ubq*	Sole UBQ-F^a^	AGCTGGCCCAGAAATATAACTGCGACA	93
	SoleUBQ-R^a^	ACTTCTTCTTGCGGCAGTTGACAGCAC	
*Rps4*	SoleRPS4-F^a^	GTGAAGAGCTCCTTGTCGGCACCA	83
	SoleRPS4-R^a^	AGGGGGTCGGGGTAGCGGATG	
*Mx*	SoleMxF^b^	CCTCTCTCCTTCAGGATCCTCCTCCTGTGC	113
	SoleMxR^b^	CAAAACAAGAAACTATCTGCCTGGTGGTTC	
*Isg15*	SoleISG15-F^c^	GATCTGAGCGACGACACCAA	150
	SoleISG15-R^c^	CTCGTCAGGTGATGTCATAGG	
*Pkr*	SolePKR-F^c^	GAGTACAAGGGAACCCCGTCTT	150
	SolePKR-R^c^	GGCATCGTCCCAAATCTGTT	
VHSV	VHSV-F1^d^	AAGGCCCTCTATGCGTTCATC	122
*nucleoprotein*	VHSV-R1^d^	GGTGAACAACCCAATCATGGT	

^a^[54]

^b^ [27]

^c^ This study, these primers have been designed according to the sequence published in the transcriptome database for Senegalese sole, SoleaDB [55]

^d^[28]

All real-time PCR reactions were performed in 20-μL mixtures containing 10 μL of 2 × Fast essential-SYBR Green PCR Master Mix (Roche), 1 μL of each primer (0.75 μM, final concentration), and cDNA (100 ng). The amplification profile was 5 min at 95 °C, followed by 40 cycles of 15 s at 95 °C, 20 s at 60 °C and 15 s at 72 °C. Fluorescence was measured at 60 °C in each cycle. Samples were run in triplicate with non-template controls on the same plate. Reactions were conducted with the LightCycler 480 II (Roche) system in 96-well plates, and data were analyzed with the LightCycler 480 software version 1.5.1. Relative cDNA levels were calculated by the 2^−∆∆CT^ method and expressed as relative fold change respect to a calibrator group, the negative control group (L15) [38, 39]. According to Livak and Schmittgen [38], mean relative fold change values ±SD <2 were considered as non-detected (ND). Relative data were log-transformed for statistical analysis. Mean values were statistically analyzed by one-way analysis of variance (ANOVA) and the Bonferroni test. Differences were considered statistically significant when *p* < 0.05.

## Results

### ISG transcription and viral genome quantification after VHSV infection

The relative transcription of *Mx*, *Isg15* and *Pkr,* as well as the viral genome, were quantified and comparatively analyzed after the inoculation of two different VHSV isolates, a marine isolate, pathogenic to Senegalese sole, and a freshwater isolate, non-pathogenic to this fish species. Poly I:C-inoculated animals were used as the positive control.

**Figure 2:**
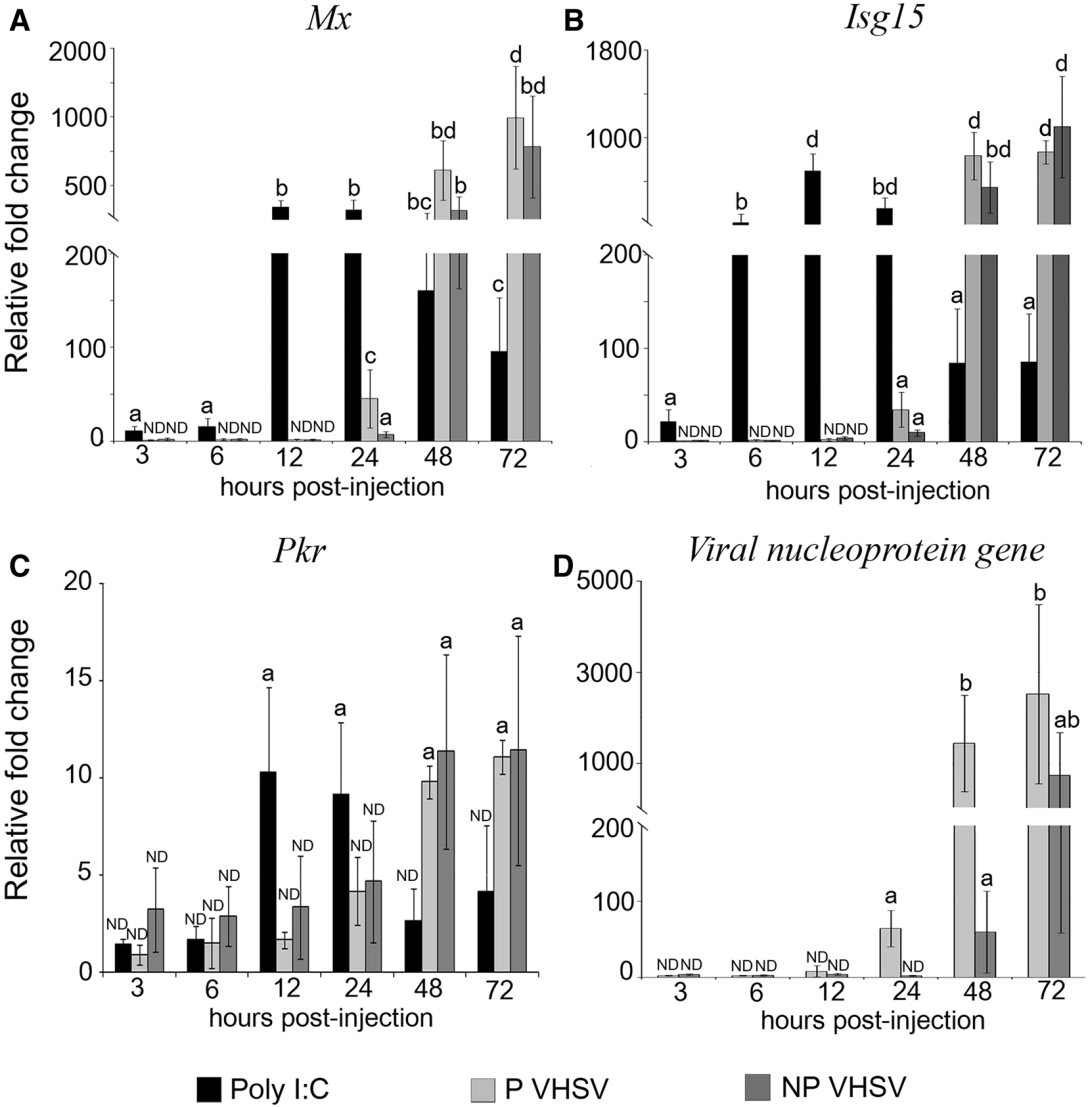
**Relative fold change of ISG transcription (A–C) and viral RNA (D) by RT-qPCR.** The study was conducted in head kidney from animals inoculated with VHSV isolates pathogenic (P) and non-pathogenic (NP) to sole. Poly I:C-inoculated animals were used as positive control. Bars indicate mean ± standard deviation (SD) obtained from three different samples. Different letters denote significant differences (*p* < 0.05) between groups at each sampling time and within the same group throughout the time. ND: Non-detected relative fold change with respect to the negative control group (L15, not represented in the graphic).

As shown in Figure 2A–C, poly I:C induces the transcription of *Mx* and *Isg15* (both from 3 to 72 hpi), as well as *Pkr* (at 12 and 24 hpi). The highest fold induction was recorded for the *Isg15* gene at 12 and 24 hpi (699 and 357 mean relative fold change values, respectively).

Both VHSV isolates also induced the transcription of the three ISG (Figure 2A–C). The P VHSV isolate induced *Mx* and *Isg15* transcription from 24 hpi onwards (Figure 2A, B), and *Pkr* transcription at 48 and 72 hpi (Figure 2C). The NP VHSV isolate stimulated *Mx*, *Isg15* and *Pkr* transcription at 48 and 72 hpi at the same level as the P VHSV (Figure 2A–C).

In order to examine viral replication, the viral nucleoprotein gene was quantified at different times post-inoculation (pi). As shown in Figure 2D, the P VHSV isolate displayed an earlier genome replication (at 24 hpi), compared to the NP VHSV (at 48 hpi). Thus, the level of viral RNA at 24 and 48 hpi was lower in animals inoculated with the NP VHSV isolate (2.33 and 59.82 mean relative fold change values, respectively) than in fish injected with the P VHSV (64.7 and 1688 mean relative fold change values, respectively); however, similar viral RNA levels were recorded for both isolates at 72 hpi.

The NP VHSV titers in pooled spleen and heart samples were stable from 3 to 72 hpi (ca. 10^4^ TCID_50_/g). The P VHSV titers were similar to those obtained with the NP VHSV from 3 to 48 hpi but increased at 72 hpi, displaying viral titers tenfold higher at the same time pi.

### Effect of the IFN I system stimulated by poly I:C on VHSV infection

In order to study the role of the IFN I system against VHSV infection, fish were inoculated with poly I:C and subsequently challenged with the marine or freshwater VHSV isolates (Figure 1B).

**Figure 3:**
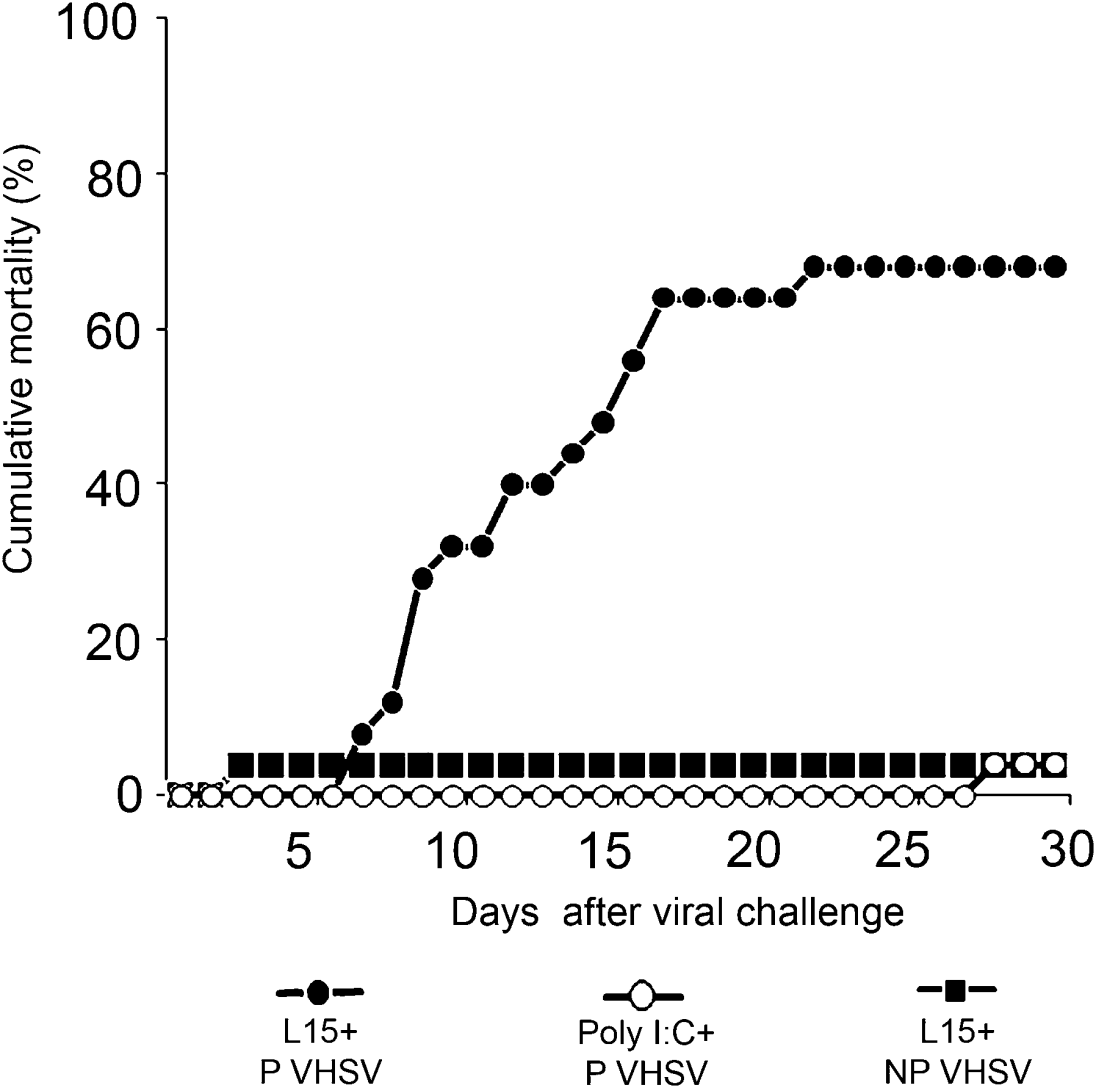
**Cumulative mortality curves.** Cumulative mortality (%) in animals inoculated with pathogenic (P) or non-pathogenic (NP) VHSV isolates. Viral inoculation was performed 24 h after the first inoculation with L-15 (L15 + VHSV groups) or poly I:C (poly I:C + VHSV group).

A drastic reduction of the mortality caused by the marine isolate was recorded in fish previously injected with poly I:C (poly I:C + P VHSV group, 5%) compared to mortality in non-stimulated animals (L15 + P VHSV group, 68%) (Figure 3). In this group, mortality onset was at 6 days post-inoculation (dpi), reaching the maximum value at 9 dpi. Mortality was stabilized at 16–17 dpi. Fish inoculated with the NP VHSV isolate (L15 + NP VHSV group) did not show any mortality (Figure 3). Infective viral particles were isolated from dead fish.

**Figure 4:**
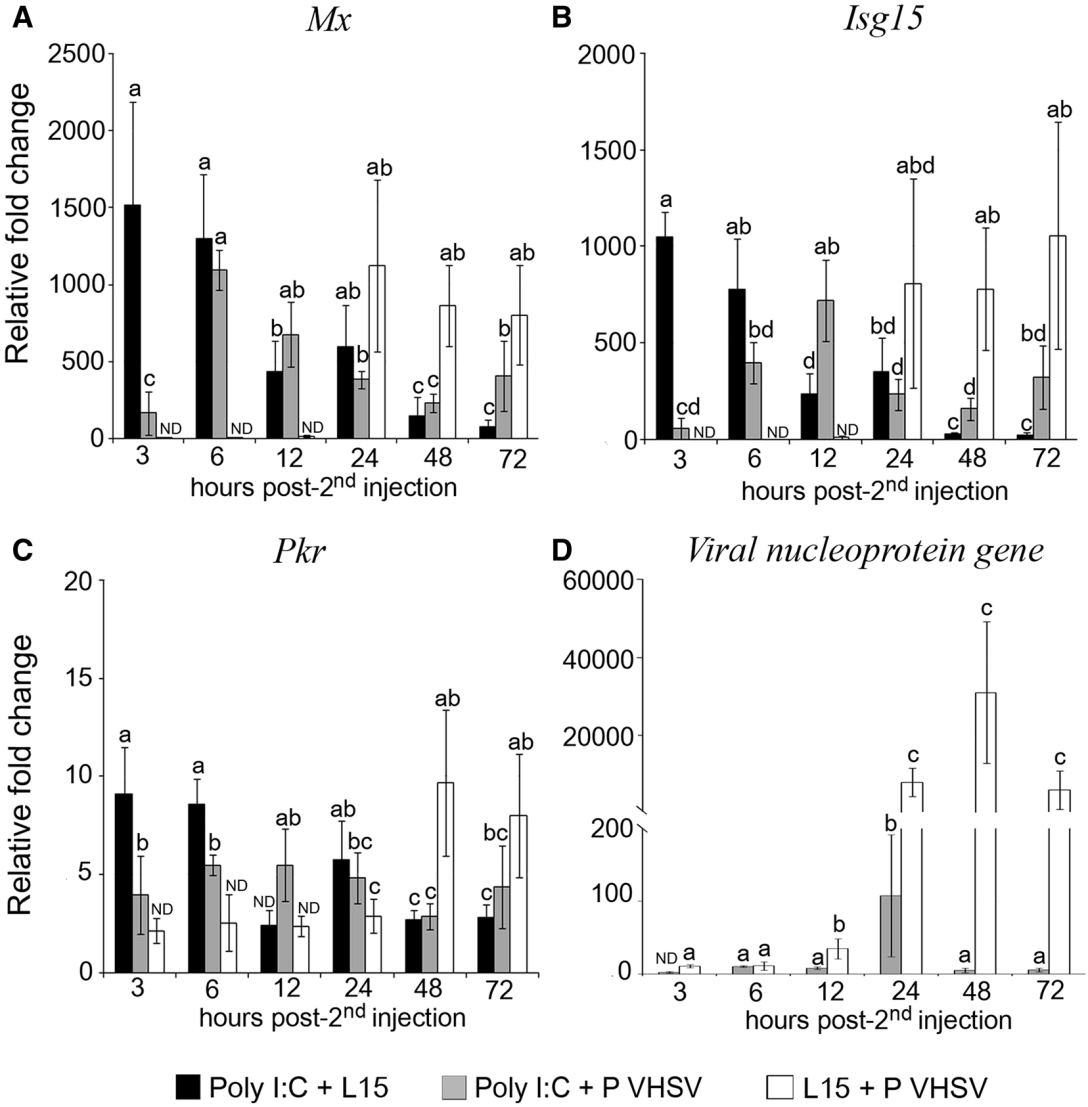
**Interaction between the IFN I system and the infection with the pathogenic (P) VHSV isolate.** The quantification of ISG transcription (**A**–**C**) and viral RNA (**D**) were evaluated in head kidney from poly I:C-stimulated and non-stimulated animals inoculated with the P VHSV isolate, and expressed as relative fold change with respect to the negative control group (L15 + L15). Poly I:C-inoculated animals were used as the positive control. Second inoculation was 24 h after the first inoculation. Bars indicate mean ± standard deviation (SD) obtained from three different samples. Different letters denote significant differences (*p* < 0.05) between groups at each sampling time and within the same group throughout the time. ND: Non-detected relative fold change with respect to the negative control group (L15 + L15, not represented in the graphic).

**Figure 5:**
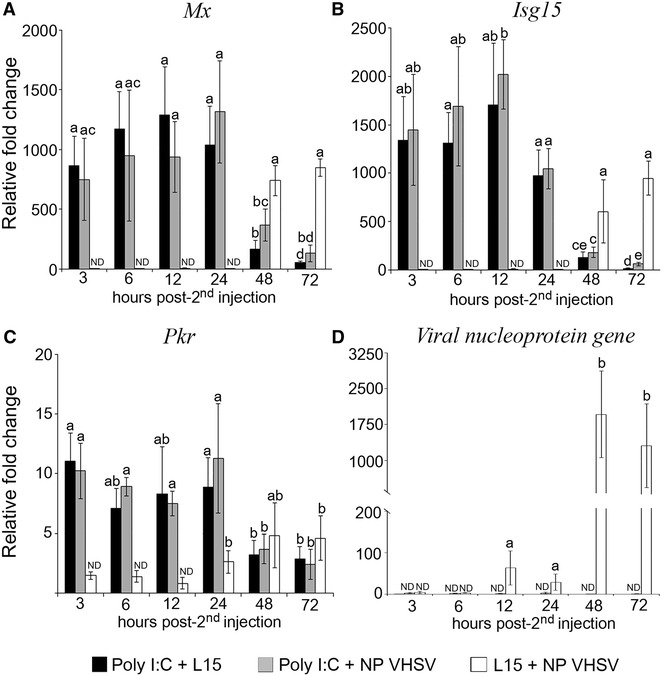
**Interaction between the IFN I system and the infection with the non-pathogenic (NP) VHSV isolate.** The quantification of ISG transcription (**A**–**C**) and viral RNA (**D**) were evaluated in head kidney from poly I:C-stimulated and non-stimulated animals inoculated with the NP VHSV isolate, and expressed as relative fold change with respect to the negative control group (L15 + L15). Poly I:C-inoculated animals were used as the positive control. Second inoculation was 24 h after the first inoculation. Bars indicate mean ± standard deviation (SD) obtained from three different samples. Different letters denote significant differences (*p* < 0.05) between groups at each sampling time and within the same group throughout the time. *ND* Non-detected relative fold change respect to the negative control group (L15 + L15, not represented in the graphic).

The stimulation of the IFN I system elicited by poly I:C resulted in an important decrease in the viral RNA fold change for both VHSV isolates at all sampling times (Figures 4D, 5D). Specifically, in fish infected with the P VHSV (L15 + P VHSV group) (Figure 4D) viral replication began at 12 hpi, although viral genome was detected from 3 hpi onwards, reaching the maximum mean value (30 000 relative fold change) at 48 hpi, and decreasing at 72 hpi. In poly I:C-stimulated fish inoculated with this isolate (poly I:C + P VHSV group), viral RNA fold change was 6200 fold lower than those recorded in non-stimulated animals at 48 hpi, when the maximum mean relative fold change value was recorded (Figure 4D). Regarding the NP VHSV replication (Figure 5D), in control fish (L15 + NP VHSV group) viral genome was detected at 12 hpi, and the maximum mean relative fold change (ca. 1000) was at 48 and 72 hpi. However, in poly I:C-stimulated animals (poly I:C + NP VHSV group) viral replication was not detected at any time tested (Figure 5D).

**Figure 6:**
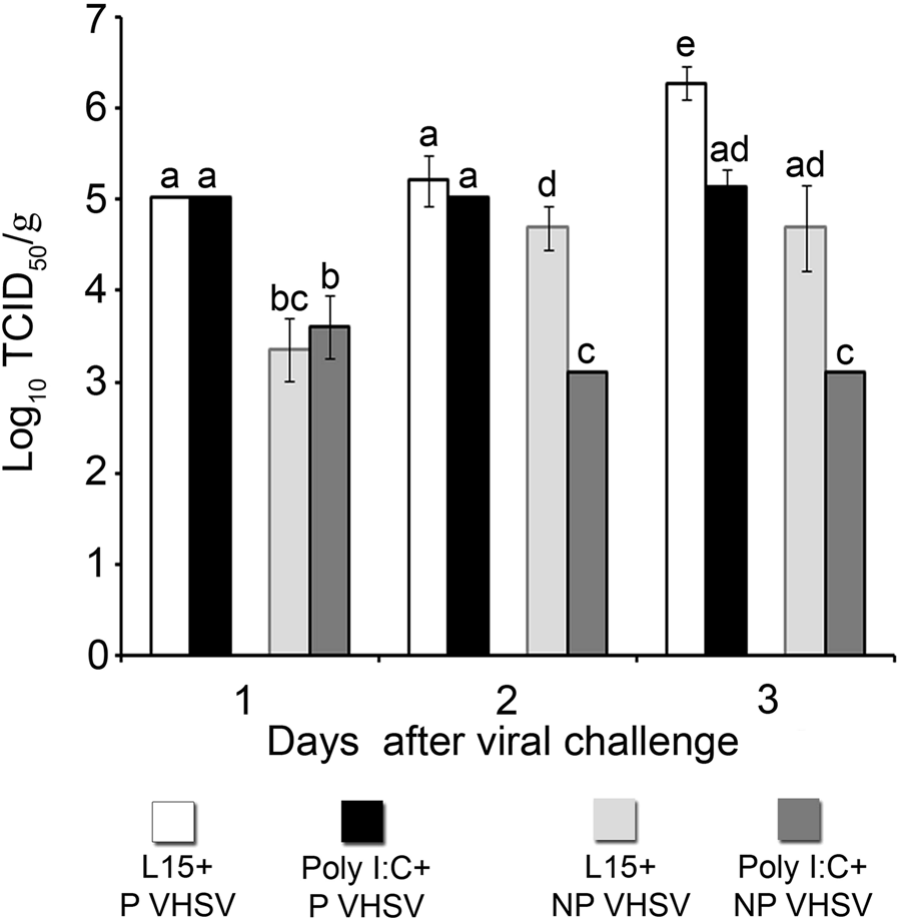
**Viral titer (log**_**10**_ **TCID**_**50**_ **/g) in pooled spleen-heart of sampled animals from the L15** **+** **VHSV and poly I:C** **+** **VHSV groups.** (P) animals inoculated with the pathogenic VHSV isolate. (NP) animals inoculated with the non-pathogenic VHSV isolate. Bars indicate mean ± standard deviation (SD) obtained from three different samples. Different letters denote significant differences (*p* < 0.05) between groups at each sampling times and within the same group throughout the time.

Infective viral particles were quantified in spleen-heart samples from infected animals (Figure 6). The P VHSV titers in stimulated fish (poly I:C + P VHSV group) remained stable throughout the time (ca. 10^5^ TCID_50_/g), whereas in control fish (L15 + P VHSV group) a significant increase (tenfold) in the viral titer was recorded at 3 days. Regarding the NP VHSV, in stimulated fish (poly I:C + NP VHSV group) titers were similar at all times tested (ca. 10^3^ TCID_50_/g), whilst in control fish (L15 + NP VHSV group) a significant increase was detected at 2 and 3 dpi (10 and 50 fold, respectively) over the average titer at 1 dpi (Figure 6).

The highest viral titers (2 × 10^6^ TCID_50_/g) were recorded from fish dead at 9 and 16 dpi (data non-shown).

### Effect of VHSV infection on the IFN I response triggered by poly I:C

The transcription of *Mx*, *Isg15* and *Pkr* was quantified in head kidney from animals injected with poly I:C and subsequently challenged with the pathogenic (Figure 4A–C) and the non-pathogenic VHSV isolates (Figure 5A–C).

In the challenge conducted with the P VHSV isolate, the fold induction of the three genes in poly I:C-stimulated animals (poly I:C + P VHSV group) was significantly lower than those recorded in the poly I:C + L15 group, mainly at 3 h post-2nd injection, for *Mx* and *Isg15*, and at 3 and 6 h post-2nd injection, for *Pkr*. However, from 12 h post viral infection onward, relative fold change values were similar in both groups or even higher in infected animals (poly I:C + P VHSV group) than in the poly I:C + L15 group (Figure 4A–C).

Regarding the NP isolate, the ISG transcription in infected and non-infected poly I:C-stimulated fish was similar at all times tested (Figure 5). As shown in Figure 5A–C, NP VHSV (L15 + NP VHSV) induces the transcription of the *Mx* and *Isg15* genes from 48 hpi and *Pkr* gene from 24 hpi. The *Isg15* and *Mx* genes showed the highest fold induction, with the maximum mean values at 72 hpi (ca. 1000 relative fold change values).

## Discussion

ISG have been classically used as indicators of the IFN I system activation by viral infections or chemical inducers, such as poly I:C. In this study, the transcription of different ISG (*Mx*, *Isg15* and *Pkr*) has been quantified in Senegalese sole after the inoculation with poly I:C or VHSV isolates with different levels of virulence, a marine isolate, pathogenic to sole under experimental conditions [26], and a freshwater isolate, which replicates in sole, although it does not cause mortality in this fish species (current study, Figure 3). This is the first report of *Isg15* and *Pkr* transcriptional induction in Senegalese sole, whereas high levels of *Mx* mRNA had been previously reported after poly I:C inoculation in this species [27].

Poly I:C inoculation resulted in the transcription of the three ISG under study, with the *Isg15* gene showing the earliest (at 3 hpi) and highest transcriptional levels, which is in concordance with previous studies. In particular, an early *Isg15* transcription has been reported in Atlantic cod (*Gadus morhua*), Japanese flounder, as well as in several fish cell lines [13, 14]. In addition, high levels of *Isg15* transcription have been recorded in Atlantic salmon (*Salmo salar*) and turbot [12, 15]. The *Mx* transcription was tenfold lower than the transcription recorded for the *Isg15* gene, although Mx transcriptional levels were similar to those recorded in other fish species such as channel catfish (*Ictalurus punctatus*), rainbow trout, Atlantic salmon, rock bream, gilthead seabream (*Sparus aurata*) and carp (*Cyprinus carpio*) [40–45]. *Pkr* transcription was 100-fold lower than *Isg15* transcription. This low transcriptional level has also been described in rock bream and fugu kidney and spleen [20, 21], and could be associated with the mechanism of PKR action, as this protein inhibits the protein synthesis, and, therefore, high levels of PKR could compromise cellular viability.

In general terms, VHSV is a strong inducer of the IFN system, stimulating the transcription of the three ISG later than poly I:C, as it was previously reported for *Mx* transcription after IPNV inoculation in Senegalese sole [27], as well as for other species infected with different viruses [40–42, 46–48], including VHSV [11]. The pattern of ISG induction after VHSV infection was similar to poly I:C stimulation: powerful induction of *Mx* and *Isg15* transcription and low *Pkr* transcription. Similar *Isg15* transcriptional levels have been described in other flatfish after VHSV infection [15, 48].

Furthermore, the kinetics and transcriptional levels of the three ISG triggered by both VHSV isolates were similar. However, the relative quantification of the viral nucleoprotein gene showed earlier and higher replication levels of the pathogenic isolate, suggesting that lower levels of RNA of the non-pathogenic VHSV isolate induce the IFN response at the same level as the pathogenic VHSV isolate. This finding might represent an important difference between both isolates regarding the interaction with the host.

The protective role of the IFN I system against VHSV infections has been evaluated in poly I:C-inoculated juvenile Senegalese sole. Animals inoculated with the non-pathogenic VHSV isolate did not show any mortality, whereas the pathogenic VHSV isolate caused 68% cumulative mortality, which is similar to mortalities previously recorded in 20-g Senegalese sole intraperitoneally inoculated with a similar dose of the same viral isolate (50% at 60 dpi, [26]). This mortality was drastically reduced (5%) by the previous poly I:C inoculation (poly I:C + P VHSV group), indicating that IFN I system stimulated by poly I:C promotes protection against VHSV infection in Senegalese sole.

In addition, the IFN I system stimulated by poly I:C compromises the multiplication of both viral isolates, as has been demonstrated by the quantification of infective viral particles and viral genome. Thus, viral titer in poly I:C-treated animals was constant throughout time, whereas in non-stimulated fish (L15 + P VHSV or L15 + NP VHSV groups) the viral titer increased up to tenfold. Furthermore, the mean viral RNA relative values were always lower in the poly I:C-treated groups than in non-stimulated fish. Previous studies have also established an antiviral state after poly I:C inoculation in several fish species against different viruses [1, 49, 50]. Regarding flatfish, it has been reported that poly I:C-treated Japanese flounder were protected against VHSV infection [51], and poly I:C inoculation of Senegalese sole decreased IPNV replication [27].

The three ISG evaluated in the current study could be involved, along with other ISG, in the antiviral state triggered by poly I:C in Senegalese sole. In fact, antiviral activity against IPNV and VHSV has been previously described for Senegalese sole Mx [28], as well as for the three ISG in other fish species. Specifically, PKR antiviral activity has been described in Japanese flounder, in which the over-expression of the *Pkr* gene inhibited *Scophtalmus maximus* rhabdovirus (SMRV) multiplication [19]; the Mx antiviral properties have been determined in several fish species [3, 11]; and the ISG15 antiviral activity has been detected in Atlantic salmon and zebrafish (*Danio rerio*) [12, 14].

The comparative analysis of the ISG transcription after viral infection in poly I:C-treated and non-treated animals revealed that the pathogenic VHSV isolate negatively interfered with the stimulation of the ISG under study at 3 and 6 hpi, whereas infection with the non-pathogenic isolate did not affect the ISG stimulation triggered by poly I:C. This result suggests that the pathogenic VHSV isolate may interfere with the sole IFN I response at early stages of infection, likely in order to evade or limit the innate host defences.

It has been previously reported that the non-structural protein (NV) is involved in the VHSV antagonistic mechanisms. In fact, this protein suppresses the activity of the Japanese flounder *Mx* gene promoter and the early activation of the nuclear factor kappa B (NF-κB) in EPC cells [52]. In addition, this viral protein also has antiapoptotic effects in EPC cells at early stages of the viral infection [53]. However, further experiments would be necessary to confirm the involvement of the NV protein in the VHSV antagonism observed in the present study.

In summary, this study has demonstrated the main role of the IFN I system against VHSV infections in Senegalese sole by several lines of evidence. The antiviral state generated by poly I:C prevents VHSV infection in juvenile Senegalese sole, reducing the cumulative mortality and the viral replication. Furthermore, the marine VHSV isolate interferes negatively with the IFN I response, affecting the transcription of the three ISG tested at early stages of viral infection. As a consequence, the marine VHSV isolate replicates earlier and at higher levels than the non-pathogenic isolate, which does not show antagonistic effect against the IFN I system. Therefore, low levels of RNA of the freshwater VHSV induced transcription of ISG to similar levels as the marine one. The differences reported for both VHSV isolates may partly explain the lack of virulence of the freshwater isolate to Senegalese sole. Further analysis of the molecular mechanisms responsible for these differences could clarify the role of viral genes and/or ISG in the interaction between VHSV and the Senegalese sole IFN I system.

## Abbreviations

ANOVA: analysis of variance
CAB: *Carassius auratus* blastulae embryonic cells
cDNA: complementary DNA
CPE: cytopathic effect
dsRNA: double-stranded RNA
elF-2α: eukaryotic initiation factor 2
EPC: epithelioma papolosum cyprini cell line
FBS: foetal bovine serum
GS: grouper spleen cells
IHNV: Infectious Hematopoietic Necrosis Virus
IFN I: type one interferon
IP: intraperitoneal
IPNV: Infectious Pancreatic Necrosis Virus
ISG: interferon stimulated gene
ISG15: interferon stimulated gene 15
L15: Leibovitz’s medium
N: nucleoprotein
ND: non-detected
NF-κB: nuclear factor kappa B
NP: non-pathogenic
NV: non-structural viral protein
P: pathogenic
pi: post-inoculation
PKR: protein kinase R
Poly I:C: polyinosinic:polycytidylic acid
qRT-PCR: quantitative reverse transcription polymerase chain reaction
Rps4: ribosomal protein S4
RTG-2: rainbow trout gonad cell line
SD: standard deviation
SMRV: *Scophtalmus maximus* rhabdovirus
SsMx: Senegalese sole Mx protein
TCID_50_: 50% tissue culture infective dose
Ubq: ubiquitin
VHS: Viral haemorrhagic septicaemia
VHSV: Viral Haemorrhagic Septicaemia Virus
NNV: Nervous Necrosis Virus
